# *IRF2BP2* Mutation Is Associated with Increased STAT1 and STAT5 Activation in Two Family Members with Inflammatory Conditions and Lymphopenia

**DOI:** 10.3390/ph14080797

**Published:** 2021-08-13

**Authors:** Maaria Palmroth, Hanna Viskari, Mikko R. J. Seppänen, Salla Keskitalo, Anniina Virtanen, Markku Varjosalo, Olli Silvennoinen, Pia Isomäki

**Affiliations:** 1 Molecular Immunology Group, Faculty of Medicine and Health Technology, Tampere University, 33520 Tampere, Finland; maaria.palmroth@tuni.fi (M.P.); anniina.t.virtanen@tuni.fi (A.V.); olli.silvennoinen@tuni.fi (O.S.); 2 Department of Internal Medicines, Tampere University Hospital, 33520 Tampere, Finland; hanna.viskari@pshp.fi; 3 Faculty of Medicine and Life Sciences, Tampere University, 33520 Tampere, Finland; 4 Rare Disease and Pediatric Research Centers, Children’s Hospital, University of Helsinki and Helsinki University Hospital, 00290 Helsinki, Finland; mikko.seppanen@hus.fi; 5 Molecular Systems Biology Group, Institute of Biotechnology, University of Helsinki, 00790 Helsinki, Finland; salla.keskitalo@helsinki.fi (S.K.); markku.varjosalo@helsinki.fi (M.V.); 6 Fimlab Laboratories, Pirkanmaa Hospital District, 33520 Tampere, Finland; 7 HiLIFE Helsinki Institute of Life Sciences, Institute of Biotechnology, University of Helsinki, 00790 Helsinki, Finland; 8 Centre for Rheumatic Diseases, Tampere University Hospital, 33520 Tampere, Finland

**Keywords:** interferon regulatory factor-2 binding protein-2 (IRF2BP2), JAK–STAT pathway, interferons, inflammation

## Abstract

Interferon regulatory factor 2 binding protein 2 (IRF2BP2) is a transcriptional coregulator that has an important role in the regulation of the immune response. IRF2BP2 has been associated with the Janus kinase (JAK)—signal transducers and activators of transcription (STAT) pathway, but its exact role remains elusive. Here, we identified a novel clinical variant, *IRF2BP2* c.625_665del, from two members of a family with inflammatory conditions and investigated the function of IRF2BP2 and c.625_665del mutation in JAK–STAT pathway activation and inflammatory signaling. The levels of constitutive and cytokine-induced phosphorylation of STATs and total STAT1 in peripheral blood monocytes, T cells, and B cells from the patients and four healthy controls were measured by flow cytometry. Inflammation-related gene expression was studied in peripheral blood mononuclear cells using direct digital detection of mRNA (NanoString). Finally, we studied the relationship between IRF2BP2 and STAT1 activation using a luciferase reporter system in a cell model. Our results show that patients having the *IRF2BP2* c.625_665del mutation presented overexpression of STAT1 protein and increased constitutive activation of STAT1. In addition, interferon-induced JAK–STAT signaling was upregulated, and several interferon-inducible genes were overexpressed. Constitutive phosphorylation of STAT5 was also found to be upregulated in CD4^+^ T cells from the patients. Using a cell model, we show that IRF2BP2 was needed to attenuate STAT1 transcriptional activity and that IRF2BP2 c.625_665del mutation failed in this. We conclude that IRF2BP2 has an important role in suppressing immune responses elicited by STAT1 and STAT5 and suggest that aberrations in IRF2BP2 can lead to abnormal function of intrinsic immunity.

## 1. Introduction

Interferon regulatory factor 2 binding protein 2 (IRF2BP2) is a transcriptional coregulator having an important role in the regulation of immune response [1]. IRF2BP2 was originally identified as a co-repressor of IRF2 [2], which counteracts IRF1 and thereby suppresses interferon signaling [3]. However, IRF2BP2 is also expressed in organisms lacking IRF2 [2], and subsequent studies have identified IRF2BP2 as a coregulator of various other transcription factors, such as VGLL4, NCOR1, ETO2, IRF2BPL, NFAT1, NRIF3, GR, and KLF2 [4,5,6,7,8,9,10,11]. In addition to the immune response, IRF2BP2 is involved in a variety of biological processes, such as apoptosis, angiogenesis, cell cycle, and cell differentiation [4,5,6,8,11,12]. The importance of IRF2BP2 is highlighted by the observation that IRF2BP2-deficient mice are rarely obtained and do not survive past 4 weeks due to severe growth retardation [5].

The Janus kinase (JAK)—signal transducers and activators of transcription (STAT) pathway constitutes a major signaling module in the immune system. JAKs are intracellular non-receptor tyrosine kinases that mediate signaling of about 60 different cytokines, hormones, and growth factors, including, but not limited to, interferons (IFN) and interleukins (IL) [13]. The JAK family consists of four members (JAK1–3 and TYK2), which transmit the cytokine-induced signal by phosphorylating the receptor and cellular signaling proteins such as STATs that act as transcription factors. Mammals encode seven STATs, namely, STAT1–4, STAT5a, STAT5b, and STAT6, which are major determinants of cytokine-specific gene responses [14]. The JAK–STAT signaling pathway is under tight regulation and is modulated by multiple regulatory proteins [15].

The clinical significance of IRF2BP2 has become evident in recent years, as familial *IRF2BP2* germline mutations causing inborn errors of immunity have been reported [16,17]. Defects in IRF2BP2 are also associated with cancer, as an *IRF2BP2*–*retinoic acid receptor alpha* (*RARA*) fusion gene and transcript has been detected in acute promyelocytic leukemia patients [18,19,20,21,22,23]. The association between IRF2BP2 and JAK–STAT signaling has been suggested previously. IRF2BP2 was shown to decrease IL-2-induced JAK–STAT5 signaling in mouse CD4^+^ T cells [24]. In addition, since IRF2 has been shown to repress IFNα and IFNβ-induced gene expression in mice [3], IRF2BP2 as a co-repressor of IRF2, could be expected to have similar role in IFNα/β signaling.

In the current study, we identified the novel variant *IRF2BP2* c.625_665del from a family of two individuals with inflammatory conditions. We examined the connection between IRF2BP2 and JAK–STAT signaling in vivo using patient samples and studied the potential effects of the clinical variant on JAK–STAT signaling pathways in a cell model.

## 2. Results

### 2.1. Clinical History

Our index patient was a 57-year-old HIV-negative male who had had severe acne and hidradenitis suppurativa requiring surgical revisions during adolescence and adulthood. At the age of 51, he underwent uncomplicated cholecystectomy and umbilical hernia repair. Soon after, he started to have subfebrile fever and fatigue, and lost up to 24 kg of weight (starting weight, 103 kg). He presented with mild lymphadenopathy, moderate splenomegaly (15 cm), and marked lymphopenia (400–650 cells/uL). Gastroscopy revealed a healing erosion in the duodenum. After thorough examinations with no other finding (colonoscopy, capsule endoscopy, bone marrow biopsy, PET-CT, HRCT) the symptoms and weight loss ceased. Three years later, the symptoms returned, and a new symptom of extensive painful oral and genital ulcers presented. He developed a deep neck abscess that was surgically drained with the extraction of a molar tooth. Ulcer problems with occasional stomachache continued. A strictured small intestine, probably leading to partial occlusion of the bowel, was noted in re-endoscopy. Biopsies from tongue and genital lesions showed unspecific ulceration with neutrophilic inflammation. Immunological analyses did not reveal antibody deficiency (Supplementary Table S1). Oral ulcers have partially responded to repeated corticosteroid injections into oral mucosa, but new lesions appear regularly.

Besides his 71-year-old sister, the index had no close living relatives. His sister had also been suffering from hidradenitis suppurativa for over 10 years and showed significant lymphopenia but no ulcer problems or a marked infection history. In addition, she has diabetes mellitus type 2, hypertension, and hyperthyroidism. At the age of 71 years, she developed difficulties keeping her head position and cramping of the arms. She was diagnosed with dystrofia myotonica type 2 with typical CCTG expansion in *CNBP* gene.

### 2.2. Family Members Possess a Heterozygous IRF2BP2 Variant

Due to unique clinical features combined with lymphopenia, the index patient was subjected to whole-exome sequencing. A novel heterozygous 41-bp deletion variant in *IRF2BP2*, c.625–665del, p. (Ala209Glnfs*31) was noted. This 41-bp deletion generates a frameshift leading to a premature stop codon at position 31 in the new reading frame. No previously established disease genes were detected for the index patient. The index patient’s sister also carried the heterozygous 41-bp deletion in *IRF2BP2*.

### 2.3. Phosphorylation of STAT1 and STAT5 Are Altered in Circulating Leukocytes of the Family Members

Since IRF2BP2 is suggested to regulate cytokine-induced JAK–STAT pathways, we studied constitutive and cytokine-induced STAT phosphorylation in circulating immune cells from the index patient and his sister using a multicolor flow cytometric assay. As the staining procedures were performed without cell purification or manipulation using fresh blood, the results are likely to reflect the phosphorylation status of STAT proteins in vivo.

**Figure 1:**
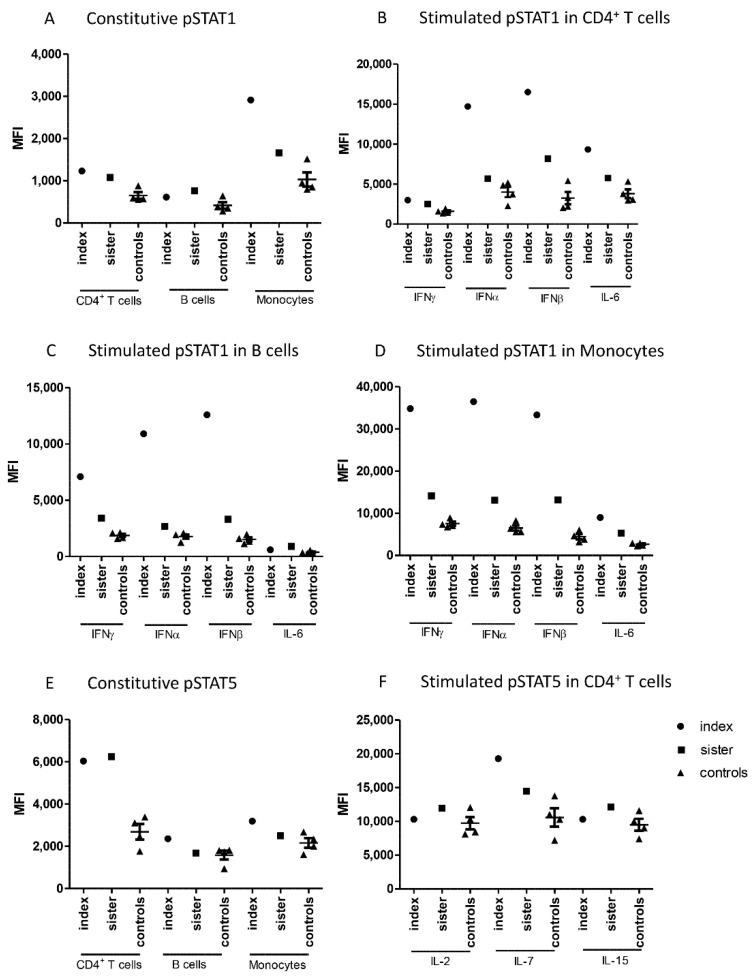
Median fluorescence intensities (MFI) measured in cells from patients and control subjects by flow cytometry for (**A**) Constitutive pSTAT1 in CD4^+^ T cells, B cells, and monocytes, (**B**) Stimulated pSTAT1 in CD4^+^ T cells, (**C**) Stimulated pSTAT1 in B cells, (**D**) Stimulated pSTAT1 in monocytes, (**E**) Constitutive pSTAT5 in CD4^+^ T cells, B cells, and monocytes (**F**) Stimulated pSTAT5 in CD4^+^ T cells. IFN; interferon, IL; interleukin, MFI; median fluorescence intensity, STAT; signal transducer and activator of transcription.

Constitutive phosphorylation of STAT1 in CD4^+^ T cells and monocytes was slightly increased in the patients when compared with healthy controls (Figure 1A). Type I and type II interferon-induced phosphorylation of STAT1 was markedly higher in the patients than in the controls in all investigated cell populations (Figure 1B–D). This difference was more prominent in the index patient than in his sister in all cell populations, and the most notable differences were seen in monocytes. IL-6-induced STAT1 phosphorylation in CD4^+^ T cells and monocytes was modestly increased in both patients.

Flow cytometric analysis showed also differences in the phosphorylation of STAT5. Constitutive STAT5 phosphorylation was significantly higher in CD4^+^ T cells from the patients compared to the controls (Figure 1E). In contrast, cytokine-stimulated STAT5 phosphorylation in CD4^+^ T cells did not generally differ between patients and controls, except for increased IL-7-induced STAT5 phosphorylation in the index patient (Figure 1F).

Constitutive as well as IFNα- and IFNβ-induced phosphorylation of STAT4 were also studied. STAT4 phosphorylation was induced by interferons only in CD4^+^ T cells, and this induction was slightly higher in the index patient than in the controls. (Supplementary Figure S1). Constitutive or cytokine-induced phosphorylation levels of STAT3 and STAT6 did not differ between family members and controls in any leukocyte population studied (Supplementary Figure S1).

### 2.4. STAT1- and IFN-Regulated Genes Are Upregulated in the Family Members

**Figure 2:**
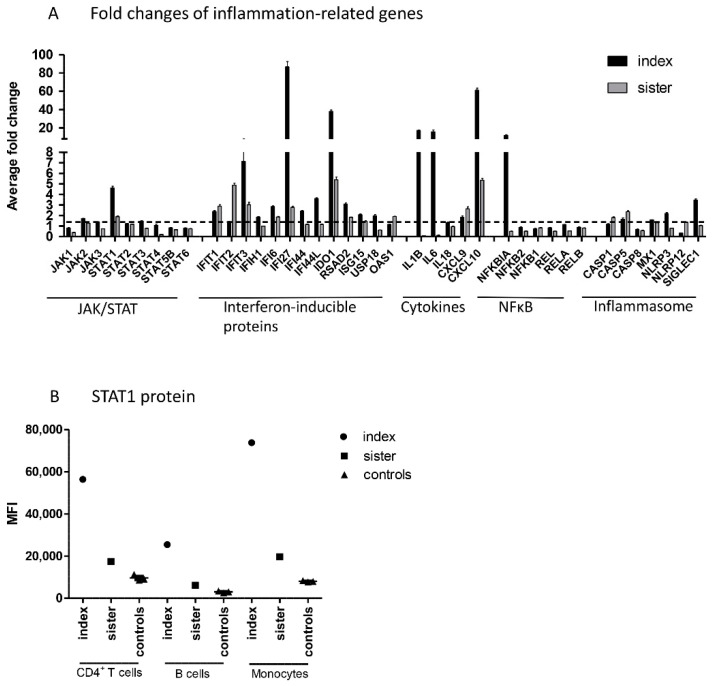
(**A**) Fold changes of inflammation-related genes in PBMCs from patients compared to those from control subjects. Inflammation-related custom gene set of the JAK–STAT pathway, interferon-inducible proteins, cytokines, NFκB pathway, and inflammasome activation were measured using the NanoString technology. Average fold changes of three technical replicates are presented. Dashed line represents a fold change of 1.5 that was considered as a threshold for increased gene expression, (**B**) STAT1 protein level in CD4^+^ T cells, B cells, and monocytes measured in cells from patients and control subjects by flow cytometry. IFN; interferon, JAK; Janus kinase, MFI; median fluorescence intensity, NFκB; nuclear factor kappa-light-chain-enhancer of activated B cells, STAT; signal transducer and activator of transcription, PBMC; peripheral blood mononuclear cell.

Next, NanoString analysis was used for direct detection of mRNA levels of JAK–STAT pathway genes, genes involved in IFN response, and other inflammation-related genes in peripheral blood mononuclear cells (PBMCs) from patients and controls. Regarding the JAK–STAT pathway, increased mRNA levels of *STAT1*, but no other *JAK* or *STAT* genes, were demonstrated in both family members (Figure 2A). Upregulation of STAT1 was also shown at protein level, as STAT1 expression was strongly upregulated in the index patient and slightly increased in his sister in all studied cell populations, using flow cytometry (Figure 2B).

In addition to *STAT1*, both family members had increased mRNA levels of interferon-inducible proteins *IFIT1*, *IFIT3*, *IFI6*, *IFI27*, *IDO1*, and *RSAD2*, cytokines *CXCL9* and *CXCL10*, and *CASP5* (10 common upregulated genes; fold change ≥1.5). In addition, the index patient, but not his sister, showed strong upregulation of *IL-1B*, *IL-6*, and *NFκBIA*. Altogether, the index patient had 12 upregulated genes that showed either normal or downregulated expression in the sister. The index patient and sister had no common downregulated genes (fold change ≤0.5).

### 2.5. IRF2BP2 Is Needed to Attenuate Constitutive and Cytokine-Induced STAT1 Transcriptional Activity

To study the relationship between IRF2BP2 and JAK–STAT pathway activation more directly, we used a dual-luciferase assay and analyzed the effects of wild-type IRF2BP2 and IRB2BP2 Δ625–665 on STAT1-mediated transcription. For this purpose, a reporter construct including an interferon-stimulated response element (ISRE-Luc) was used.

**Figure 3:**
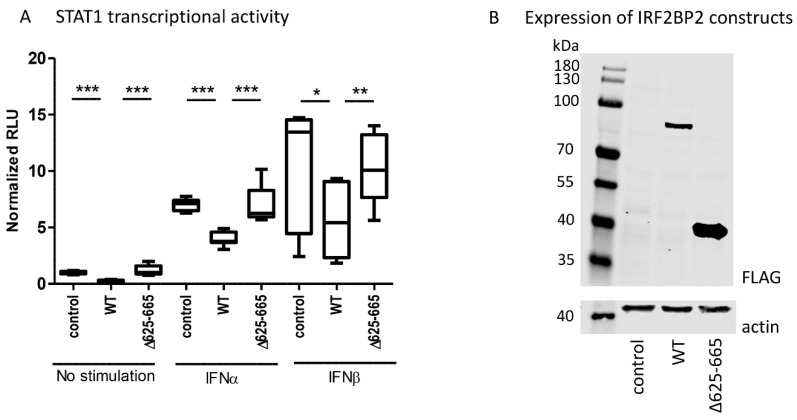
(**A**) The effect of wild-type IRF2BP2 and IRF2BP2 Δ625-665 on STAT1 transcriptional activity was studied using the ISRE-Luc reporter in HEK293T cells. (**B**) Detection of wild-type IRF2BP2 and IRF2BP2 Δ625-665 expression in HEK293T cells by immunoblotting. Statistical analyses were performed on three independent experiments, and Student’s two-tailed T-test was used to calculate significant differences. Significant differences are marked with an asterisk: * *p* ≤ 0.05, ** *p* ≤ 0.01, *p* *** ≤ 0.001. IFN; interferon, IRF2BP2; interferon regulatory factor 2 binding protein 2, RLU; relative luciferase unit, STAT; signal transducer and activator of transcription.

The reporter assay showed that wild-type IRF2BP2 significantly suppressed constitutive and cytokine-stimulated STAT1 transcriptional activity in HEK293T cells, when compared to control (Figure 3A). Importantly, IRF2BP2 Δ625–665 was not able to suppress the transcriptional activity. The expression of wild-type and Δ625–665 IRF2BP2 in HEK293T cells was shown by immunoblotting (Figure 3B).

## 3. Discussion

In this study, we describe a novel variant of *IRF2BP2*, c.625_665del p.(Ala209Glnfs*31), which was present in two members of a family with inflammatory conditions and lymphopenia. The patients demonstrated increased levels of STAT1 at both mRNA and protein levels, as well as increased constitutive and cytokine-induced STAT1 phosphorylation in circulating leukocytes. In addition, constitutive STAT5 phosphorylation was increased in CD4^+^ T cells. We also demonstrate that wild-type IRF2BP2, but not IRF2BP2 Δ625–665, attenuated STAT1 transcriptional activity in a cell model.

The IRF2BP2 protein is an 87 kDa protein consisting of 587 amino acids. It contains two conserved regions: an N-terminal zinc finger and a C-terminal RING domain [2]. The zinc finger typically binds to specific DNA sequences [25], while the RING domain has been shown to be essential in protein–protein interactions formed by IRF2BP2 in transcriptional repressor complexes [2,5,6]. In addition, RING domain-containing proteins play a crucial role in transferring ubiquitin to substrate proteins or other RING domain-containing proteins [26].

Until now, germline *IRF2BP2* mutations have been reported in two families [16,17], this study reporting the third family and mutation described. Keller et al. identified a heterozygous point mutation in *IRF2BP2* (c.1652G > A: p. (S551N)) from three members of a family diagnosed with common variable immunodeficiency disorder (CVID) and showed in vitro that this variant decreased B cell maturation into plasmablasts [16]. Baxter et al., in turn, discovered a heterozygous *IRF2BP2* variant (c.1606insTTT, p.Q536delinsX) that produces a truncated protein at position 536 of 587. This variant was suggested as one of the candidate mutations in childhood-onset immune dysregulation, the patient having a similar phenotype as patients reported by Keller et al. [17]. The clinical variant described in the current study generates a truncated protein of only 238 amino acids of 587 and entirely lacks the C-terminal RING domain. Thus, all three described clinical *IRF2BP2* variants affect the RING domain. Despite the low patient numbers, interestingly, the patient phenotype in the current study is more inflammatory than immunodeficient, as the patients did not present recurrent infections, in contrast to patients in earlier studies [16,17]. Mucosal ulcers in the index patient together with increased *CASP5* transcription resemble both the first described systemic non-canonical inflammasomopathy due to a neomorphic *CEBPE* mutation and Behçet disease [27] and require further study in a larger cohort.

IRF2BP2 was first described as a co-repressor of IRF2 [2]. IRF1 and IRF2 are transcription factors that have been shown to compete in binding to the same DNA-binding elements, interferon-stimulated response elements (ISRE), and are activated in response to type I interferons (IFNs). However, they have opposite functions: IRF1 activates IFN-induced gene expression, while IRF2 represses it [28,29]. IRF1 has also been shown to be as essential as STAT1 in activating IFNγ-induced gene expression [30], suggesting a role for IRF2 also in type II IFN signaling.

**Figure 4:**
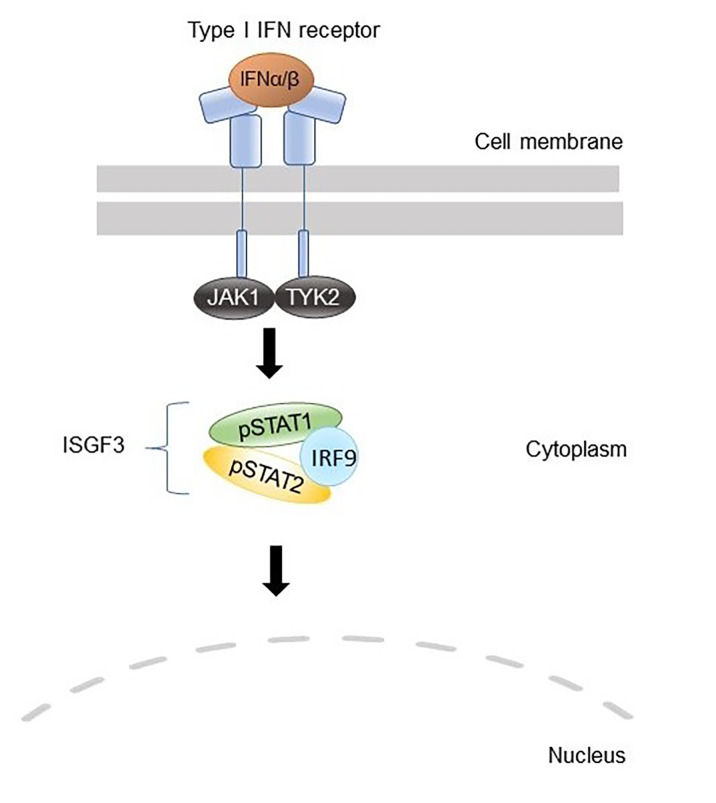

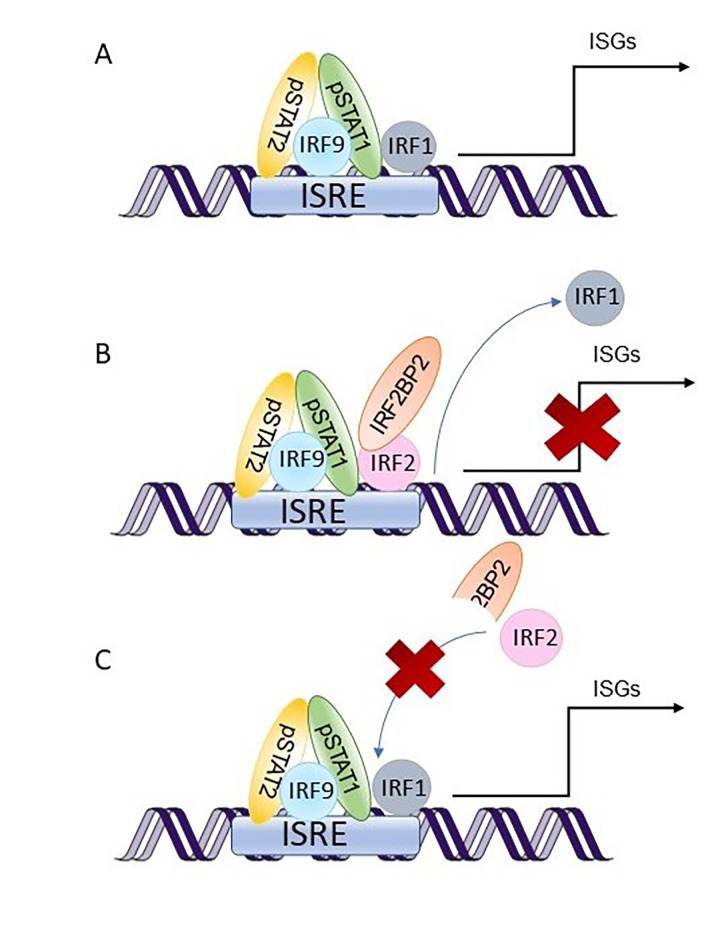
A schematic picture illustrating the suggested role of IRF2BP2 in type I interferon (IFN) signaling. IFN binding to their receptors activates JAKs, which phosphorylate STATs. Upon IFNα or IFNβ stimulation, the transcription factor complexes called interferon-stimulated gene factor 3 (ISGF3) that consist of STAT1, STAT2, and interferon regulatory factor 9 (IRF9) are formed. These transcription factor complexes translocate to the nucleus and bind to interferon-stimulated response elements (ISRE). (**A**) IRF1 is a transcription factor that co-operates with ISGF3 to induce interferon-stimulated genes (ISGs). (**B**) IRF2 restricts the induction of ISGs by competing with IRF1 for the same binding site. IRF2BP2 acts as a co-repressor of IRF2 by binding to it through the C-terminal RING domain and is essential for IRF2 ability to limit the expression of ISGs. (**C**) Patient mutation *IRF2BP2* c.625_665del generates a truncated IRF2BP2 protein (238 amino acids of 587) that entirely lacks the RING domain. Truncated IRF2BP2 cannot bind to IRF2, which in turn is unable to bind DNA and suppress IRF1 function. IFN; interferon, IRF; interferon regulatory factor, IRF2BP2; interferon regulatory factor 2 binding protein 2, ISGF3; interferon-stimulated gene factor 3, ISRE; interferon-stimulated response element, JAK; Janus kinase, STAT; signal transducer and activator of transcription.

Our data showed a systematic hyperactivation of the IFN–JAK–STAT1 axis, as the patients had increased constitutive STAT1 phosphorylation in T cells and monocytes as well as increased IFN-induced STAT1 phosphorylation in all leukocyte subtypes studied. In addition, several type I IFN inducible genes, such as *IFIT1*, *IFIT3*, *IFI6*, and *IFI27*, as well as the type II IFN-induced genes *IDO1*, *CXCL9*, and *CXCL10*, were overexpressed in PBMC from the patients. Finally, a reporter assay including the ISRE binding site showed that IRF2BP2 has a repressive role in STAT1 transcriptional activity. Taken together, our data regarding type I IFN signaling are consistent with the prevailing hypothesis of IRF2BP2 being a co-repressor of IRF2 and suggest that the increased STAT1 activation observed in the patients could be explained by the lack of suppressive effects on IRF1 function. The suggested roles of wild-type and Δ625–665 variants of IRF2BP2 in the type I IFN signaling pathway are illustrated in Figure 4.

Since gamma-activated sequences (GAS) recognized by IFNγ-induced STAT1 dimers do not contain binding sites for IRF1 [31], the mechanism how IRF2BP2 regulates type II IFN signaling probably differs from that involved in type I IFN signaling. IRF1 has been shown to form heterodimers with STAT1 and regulate the expression of *LMP2*, whose promoter includes a GAS binding site [32], but also to promote STAT1 binding to GAS without direct interaction [33]. Thus, IRF2BP2 function in type II IFN signaling might also include IRF1/2. However, more mechanistic data are still needed to confirm whether the repressive function of IRF2BP2 on STAT1 activation and IFN-induced gene expression occurs via IRF1/2.

The patients in the current study had increased levels of STAT1, both at mRNA and at protein level. Interestingly, IRF1 has been shown to induce the expression of STAT1 and pSTAT1 during viral infection [34]. The expression of both STAT1 and IRF1 is induced upon IFN stimulation [30]. This suggests that the described IRF2BP2 variant indirectly upregulates pSTAT levels: the increased constitutive and IFN-stimulated pSTAT1 levels in the patients were likely to result from overexpression of STAT1, as the induction of IFN-stimulated genes is not limited by the IRF2–IRF2BP2 complex.

IRF2BP2 has been associated with STAT5 function in a previous study concerning the role of IRF2BP2 in CD4^+^ T cell activation [24]. Sécca et al. demonstrated that overexpression of IRF2BP2 in mouse CD4^+^ T cells impaired IL-2-induced STAT5 activation and thereby restricted IL-2 high-affinity receptor α-chain expression and CD4^+^ T cell proliferation [24]. Interestingly, our data showed that both patients had increased constitutive STAT5 phosphorylation levels in CD4^+^ T cells compared to healthy controls, but not in other cell types studied. This finding supports the hypothesis of IRF2BP2 being a repressor of CD4^+^ T cell activation. However, the mechanism by which IRF2BP2 mutation affects STAT5 activation remains elusive and requires further studies.

Besides widening our knowledge on the mechanisms behind immunological diseases, studying immune responses in patients with novel genetic variants may provide clues to possible effective therapies. Our current results suggest that patients with loss-of-function IRF2BP2 mutations might benefit from treatments that inhibit interferon signaling. Finding an effective type I interferon antagonist therapy for systemic lupus erythmatosus (SLE) patients has been a major area of interest in recent years, as SLE patients show increased circulating type I interferon levels as well as overexpression of IFN-induced genes [35]. Anifrolumab, an antibody which blocks IFNAR1 receptor, is the first approved IFN antagonist therapy and received FDA approval in July 2021 [36]. Another alternative for inhibiting interferon signaling would be to block the signaling cascade one step further by using JAK inhibitor treatment. JAK inhibitors are small molecular kinase inhibitors that already include several approved inhibitors for rheumatologic, dermatologic, hematologic, and gastrointestinal indications, as well as emergency authentication for COVID-19 [37]. Given that also STAT5 was activated in T cells from patients in the current study, JAK inhibitors, that have the potential to inhibit multiple JAK–STAT pathways simultaneously, might provide a more potent therapeutic alternative than IFN antagonists for patients with loss-of-function *IRF2BP2* mutations.

The studied family had only two patients with *IRF2BP2*, c.625_665del, and the results should be considered preliminary. However, since similar effects of the *IRF2BP2* variant on STAT activation were demonstrated both with patient samples and using a cell model, the repressive role of IRF2BP2 on JAK–STAT signaling pathways seems credible. Therefore, the current study significantly adds to our knowledge of the regulatory function of IRF2BP2 in cytokine signaling.

## 4. Materials and Methods

### 4.1. Patient Samples

A family of two individuals (57-year-old index male and his 71-year-old sister) with inflammatory conditions were enrolled in the present study. Further, 3 healthy male subjects (54, 56, and 58 years of age) and 1 healthy female subject (72-year-old) were enrolled as controls. Blood samples were drawn at the same time for all the analyses, except for genetic studies that were performed separately.

### 4.2. Genetic Studies

Whole-exome test targeting all protein-coding exons, exon–intron boundaries (±20 bps), and selected clinically relevant non-coding variants was performed for both index patient and his sister. The analysis was performed using the Illumina sequencing system. Quality controlled sequence reads of each sample were mapped to the human reference genome (GRCh37/hg19), and reads were aligned using the Burrows–Wheeler Aligner (BWA-MEM). Alignment post-processing and variant calling were performed using GATK algorithms (Sentieon).

The pathogenic potential of variants was predicted by taking into account the predicted consequence, biochemical properties of the codon change, degree of evolutionary conservation, as well as allelic frequencies from large population studies (1000 Genomes project, gnomAD) and mutation databases (HGMD, ClinVar), as well as an in-house variant database.

### 4.3. Flow Cytometry

Constitutive and cytokine-induced STAT phosphorylation and level of total STAT1 were studied with multicolor flow cytometric analysis in circulating immune cells. First, 50 µL aliquots of fresh blood samples were either left unstimulated or stimulated with 100 ng/mL recombinant IFN-β, IFN-γ, IL-2, IL-4, IL-6, IL-7, IL-15, IL-21 (all from Peprotech, Rocky Hill, NJ, USA), IFN-α (Cell Signaling Technology, Danver, MA, USA), or IL-10 (R&D Systems, Minneapolis, MN, USA) for 15 min at 37 °C. The stimulation was terminated by transferring the samples on ice. Leukocytes were then fixed, and red blood cells lysed using BD Phosflow Lyse/Fix buffer (Becton, Dickinson and Company (BD), Franklin Lakes, NJ, USA) for 10 min at 37 °C, washed with PBS, and permeabilized in ice-cold methanol for 10 min on ice followed by 2–3 days at −80 °C. Following two washes with PBS, samples were stained with fluorescein isothiocyanate (FITC)-conjugated anti-CD3 (clone SK7), allophycocyanin (APC)-conjugated anti-CD33, phycoerythrin-cyanin 7 (PE-Cy7)-conjugated anti-CD20 (clone H1(FB1)) (all from BD), APC-eFluor 780-conjugated anti-CD4 (clone SK3) (eBioscience, Santa Clara, CA, USA), phycoerythrin (PE)-conjugated STAT1 (clone 1/Stat1), phospho-STAT1 (clone 4a), phospho-STAT3 (clone 4/P-STAT3), phospho-STAT4 (clone 38/p-Stat4), phospho-STAT5 (clone 47), or phospho-STAT6 (18/P-Stat6) (all from BD) for 30 min at room temperature.

**Figure 5:**
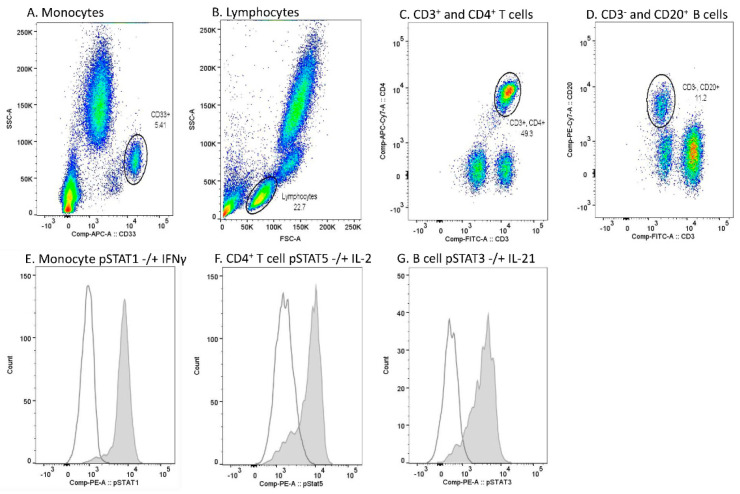
Flow cytometry gating strategy and examples of cytokine-induced phosphorylated STAT (pSTAT) in each cell population in one sample. (**A**) Monocyte gate was set based on CD33 positivity and light scattering characteristics (SSC-A). (**B**) Lymphocytes were gated based on light scattering characteristics (FSC-A and SSC-A). (**C**) Among lymphocytes, the CD4^+^ T cell gate was set to comprise CD3^+^ and CD4^+^ populations. (**D**) Among lymphocytes, the B cell gate was set to comprise CD3^−^ and CD20^+^ populations. (**E**) pSTAT1 histograms in unstimulated (open peak) and IFN-γ-stimulated (grey peak) monocytes. (**F**) pSTAT5 histograms in unstimulated (open peak) and IL-2-stimulated (grey peak) CD4^+^ T cells. (**G**) pSTAT3 histograms in unstimulated (open peak) and IL-21-stimulated (grey peak) B cells.

Data acquisition was performed using FACS Canto II (BD), and the analysis of flow cytometer data using Flowjo Single cell analysis software (BD). Monocytes were gated based on CD33 positivity and light scattering characteristics (SSC-A) (Figure 5A). Lymphocytes were gated based on light scattering characteristics (SSC-A and FSC-A) (Figure 5B). Among lymphocytes, CD4^+^ T cells were gated from the CD3^+^ lymphocyte population, and CD20^+^ B cells from the CD3^-^ lymphocyte population (Figure 5C,D). A PE fluorescence histogram was created for each cell population, and the median fluorescence intensity (MFI, arithmetic median) was calculated. Examples of cytokine-induced pSTATs in different cell populations are presented in Figure 5E–G.

### 4.4. Peripheral Blood Mononuclear Cell Isolation and RNA Extraction

PBMCs were isolated by BD Vacutainer^®^ CPT™ Mononuclear cell preparation tubes by density-gradient centrifugation and washed twice with PBS. Cells were snap-frozen without media and stored at −80 °C until RNA extraction. Total RNAs were isolated from PBMCs using the Rneasy Mini-Kit (Qiagen, Valencia, CA, USA), according to the manufacturer’s instructions.

### 4.5. Nanostring Analysis

A custom gene panel (50 genes) containing genes involved in JAK–STAT pathway, interferon-inducible proteins, cytokines, NFκB pathway, and inflammasome activation was studied using NanoString analysis. In this analysis, patient data were compared to one gender- and age-matched control (for the index patient, a 56-year-old healthy male subject, and for his sister, a 72-year-old healthy female subject).

NanoString has been described in detail earlier [27]. Shortly, 100 ng of total RNA per sample were mixed with 5′ reporter probes, tagged with fluorescent barcodes of targeted genes, and 3′ biotinylated capture probes. The samples were hybridized overnight at 65 °C following the manufacturer’s protocol. Then, the reactions were purified and immobilized on the sample cartridge surface. The cartridge was scanned in triplicate using the nCounter Digital Analyzer (NanoString Technologies, Seattle, WA, USA). Gene expression data were analyzed using nSolver™ 4.0 analysis software (NanoString Technologies). Normalized gene counts from three replicates were further used to calculate the fold changes between the patients and the controls. Genes, whose fold changes exceeded the limit of detection are presented in the manuscript.

### 4.6. Plasmid Constructs and Mutagenesis

The full-length IRF2BP2 expression plasmid (IRF2BP2-pDEST-C1-FLAG-GFP11-GW) was a kind gift from Professor Jorma Palvimo from the University of Eastern Finland [38]. The IRF2BP2 Δ625–665 plasmid construct possessing the patient’s mutation was created by QuikChange (AgilentTechnologies, Santa Clara, CA, USA) site-directed mutagenesis, according to the manufacturer’s instructions and verified by Sanger sequencing. An empty backbone expression vector was used as a control plasmid.

For luciferase reporter assays, firefly luciferase reporter constructs for STAT1 (interferon-stimulated response element-Luc (ISRE-Luc)) [39] were used together with a plasmid constitutively expressing renilla luciferase.

### 4.7. Mammalian Cell Culture

Human embryonic kidney cells (HEK293T) were cultured in Dulbecco modified Eagle medium (Lonza, Basel, Switzerland) supplemented with 10% FBS (Sigma, St Louis, MO, USA), 2 mmol/L L-glutamine (Lonza), and antibiotics (0.5% penicillin/streptomycin; Lonza). For dual luciferase assay and immunoblotting experiments, 120,000 or 100,000 cells were seeded on 12-well plates, respectively. Transient transfections were done using FuGENE HD (Promega, Madison, WI, USA) according to the manufacturer’s instructions.

### 4.8. Dual Luciferase Assay

In total, 10 ng of control plasmid, IRF2BP2 wild-type plasmid, or IRF2BP2 Δ625–665 plasmid was co-transfected with 220 ng ISRE-Luc and 60 ng renilla luciferase plasmids. Then, after 24 h of transfection, cells were transferred to a 96-well plate. After 3–4 h, when the cells had attached to the bottom, the medium was changed to starvation medium (culture medium without FBS) or stimulation medium (starvation medium supplemented with 100 ng/mL IFNα or 10 ng/mL IFNβ). Luciferase activity was measured after 20 h of starvation/stimulation. Each experiment involved three technical replicates per sample, and three independent experiments were performed. Statistical analyses were performed using the measured values for unstimulated control plasmid as a normalizer for each independent experiment. Student’s two-tailed T-test was used to calculate significant differences.

### 4.9. Immunoblotting

HEK293T cells were transfected with 100 ng of control plasmid, IRF2BP2 wild-type plasmid, or IRF2BP2 Δ625–665 plasmid. After 48 h of transfection, cell lysates were prepared for immunoblotting: cells were transferred on ice and washed with cold phosphate buffered saline (PBS). Triton X-100 lysis buffer with protease and phosphatase inhibitors (2 mM vanadate, 1 mM phenylmethanesulfonyl fluoride, 8.3 µg/mL aprotinin, and 4.2 µg/mL pepstatin) was used to lyse the cells. Then, whole cell lysates were spun for 20 min at 16,000 g, and total protein amount in the cell lysates was determined from the supernatants by the Bradford assay (BioRad). Equal amounts of total protein (30 µg) were loaded on 4–15% Mini-PROTEAN^®^ TGX™ Precast Gels (BioRad, Irvine, CA, USA).

Immunoblots were blocked with bovine serum albumin (BSA) and incubated with primary antibody for FLAG Tag (1:1,000, F1804, clone M2, Merck, Kenilworth, NJ, USA) or actin (1:2,000, MAB1501, Merc), which was used as a loading control, and with a mixture of goat anti-rabbit and goat anti-mouse DyLight secondary antibodies (both from Thermo Fisher Scientific, Waltham, MA, USA). Blots were scanned with an Odyssey CLx (LI-COR Biosciences, Lincoln, NE, USA).

## Data Availability

The data underlying this article are available in the article and in its online Supplementary Material.

## Supplementary Materials

The following are available online at https://www.mdpi.com/article/10.3390/ph14080797/s1, Supplementary Table S1: Clinical parameters of index patient and his sister, Supplementary Figure S1: Constitutive and cytokine-induced STAT3, 4, and 6 phosphorylation in CD4^+^ T cells, B cells, and monocytes.
