# Supplementary material for: IRF2BP2 Mutation Is Associated with Increased STAT1 and STAT5 Activation in Two Family Members with Inflammatory Conditions and Lymphopenia
Source: Pharmaceuticals (Basel). 2021 Aug 13;14(8):797. doi: 10.3390/ph14080797 (PMC8402006; doi:10.3390/ph14080797)
Supplement: Supplementary file 1 [file pharmaceuticals-14-00797-s001.zip › pharmaceuticals-1304946-supplementary.pdf]

Supplementary material

Palmroth et al: IRF2BP2 mutation is associated with increased STAT1 and STAT5 activation in two family members with inflammatory conditions and lymphopenia

Supplementary Table S1: Clinical parameters of index patient and sister. Values differing from reference values are marked in bold.

	Index patient: Male		Sister: Female	
Clinical findings and symptoms	Oral and genital ulcers, Hidradenitis suppurativa, Acne, Periodic abdominal pain and fever, Weight loss		Hidradenitis suppurativa, Diabetes mellitus type 2, Hypertension, Hyperthyreosis, Dystrofia myotonica 2	
Age at sampling (years)	57		71	
		Normal range (males)		Normal range (females)
Leukocytes (cells/ul)	**2000**	3400-8200	5000	3400-8200
Lymphocytes (cells/ul)	**650**	1200-3500	**470**	1200-3500
Neutrophils (cells/ul)	**1290**	1600-6200	4080	1600-6200
Platelets (cells/l)	154	150 -360	272	150 -360
Hemoglobin (g/l)	**122**	134-167	140	117-155
CD4 (cells/ul)	**230**	520-1470	ND	
CD8 (cells/ul)	**190**	210-920	ND	
CD19 (cells/ul)	**40**	90-510	ND	
CD16/56 (cells/ul)	120	70-560	ND	
IgG g/l	12,6	6,8-15,0	**4,5**	6,8-15,0
IgA g/l	3,28	0,88-4,84	ND	
IgM g/l	0,79	0,36-2,59	0,59	0,47-2,84
IgE kU/l	12	0-100	ND	
Complement activation	normal		ND	
Polysaccaride vaccine response (pneumococcal)	normal		ND	

CD; cluster of differentiation, Ig; immunoglobulin, ND; no data

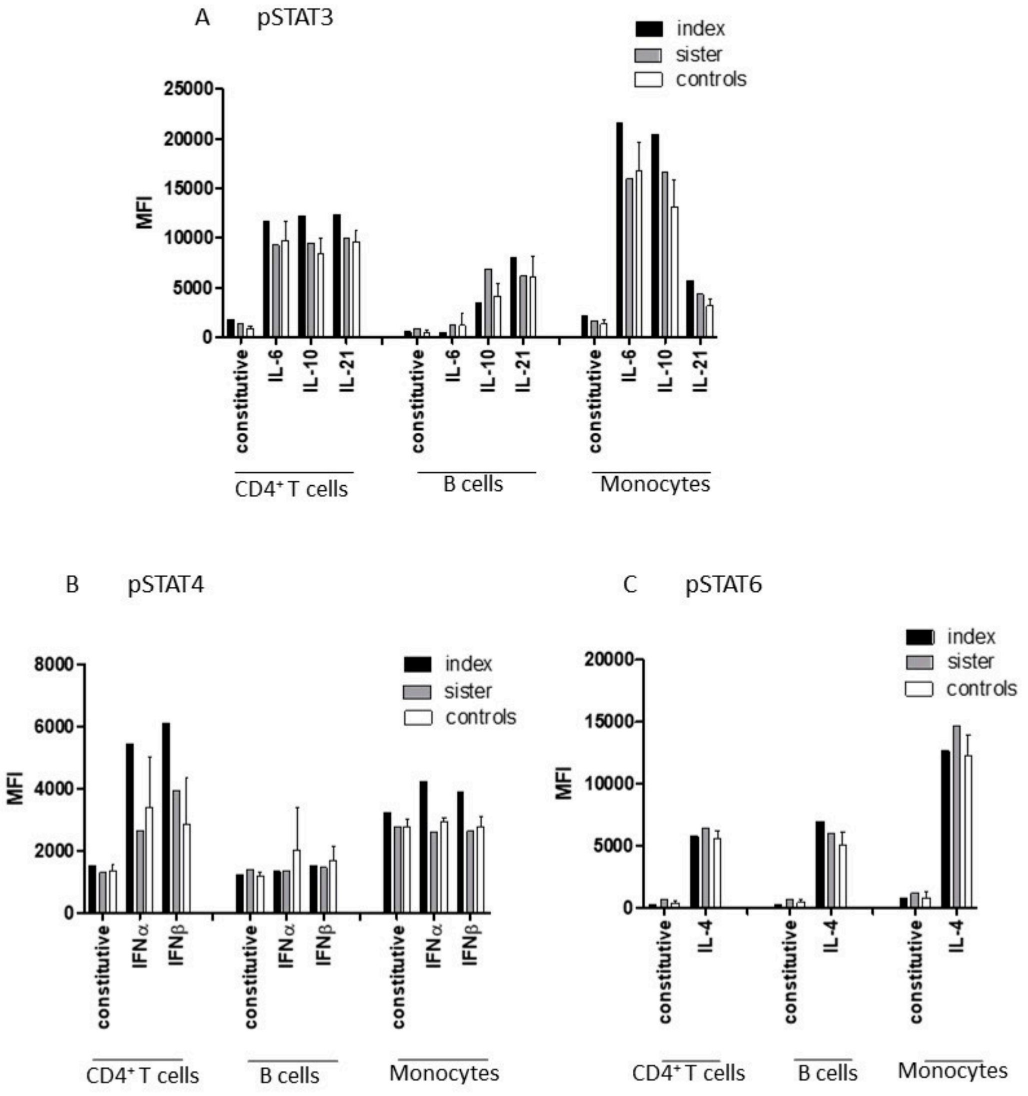

Supplementary Figure S1: Constitutive and cytokine-induced phosphorylation of A) STAT3, B) STAT4 and C) STAT6 in $CD4^{+}$ T cells, B cells and monocytes. CD; cluster of differentiation, IFN; interferon, IL; interleukin, MFI; median fluorescence intensity, STAT; signal transducer and activator of transcription.
